# Subareolar breast abscess in male patients: a report of two patients with a literature review

**DOI:** 10.1186/s40792-017-0402-3

**Published:** 2017-12-19

**Authors:** Takashi Kazama, Isao Tabei, Chikako Sekine, Naotake Funamizu, Shinji Onda, Tomoyoshi Okamoto, Hiroshi Takeyama, Toshiaki Morikawa

**Affiliations:** 1 Department of Surgery, Jikei University Daisan Hospital, 4-11-1, Izumihoncho, Komae City, Tokyo 201-8601 Japan; 2 Department of Surgery, Kawaguchi Municipal Medical Center, Saitama, Japan; 3 Department of Respiratory, Breast and Endocrine Surgery, Jikei University School of Medicine, Tokyo, Japan

**Keywords:** Breast abscess, Breast tumor, Duct resection, Smoking, Recurrence, Fine needle aspiration, Retro-areolar, Zuska’s disease

## Abstract

**Background:**

Subareolar breast abscess (SBA) is a rare infectious disease of the breast in male patients.

**Case presentation:**

Herein, we report two male patients with SBA. Patient 1 was initially diagnosed with a malignant tumor based on imaging findings; ultrasonography revealed a hypoechoic mass with blood flow. Patient 2 was diagnosed with inflammatory changes to his nipple; ultrasonography findings supported the diagnosis with an irregular hypoechoic mass with blood flow. Both patients received a cytological or histological biopsy preoperatively, which showed an abscess without malignant cells.

**Conclusion:**

These cases serve as an important reminder to consider complete resection of the tumor including the responsible mammary duct (tumor and duct resection (TDR)) for curative therapy of SBA.

## Background

A subareolar breast abscess (SBA) is an infected lump that occurs in the subareolar area. In 1951, Zuska et al. [1] first reported SBA, which is also known as Zuska’s disease, as lactiferous fistulas. SBA has some properties that are recurrent and can lead to the formation of intractable fistulas. Although SBA is frequently encountered in female patients, it rarely occurs in male patients. To our knowledge, only nine case reports of SBA in male patients have been published to date. We herein describe two patients of SBA and provide a review of the literature.

## Case presentation

### Patient 1

**Fig. 1:**
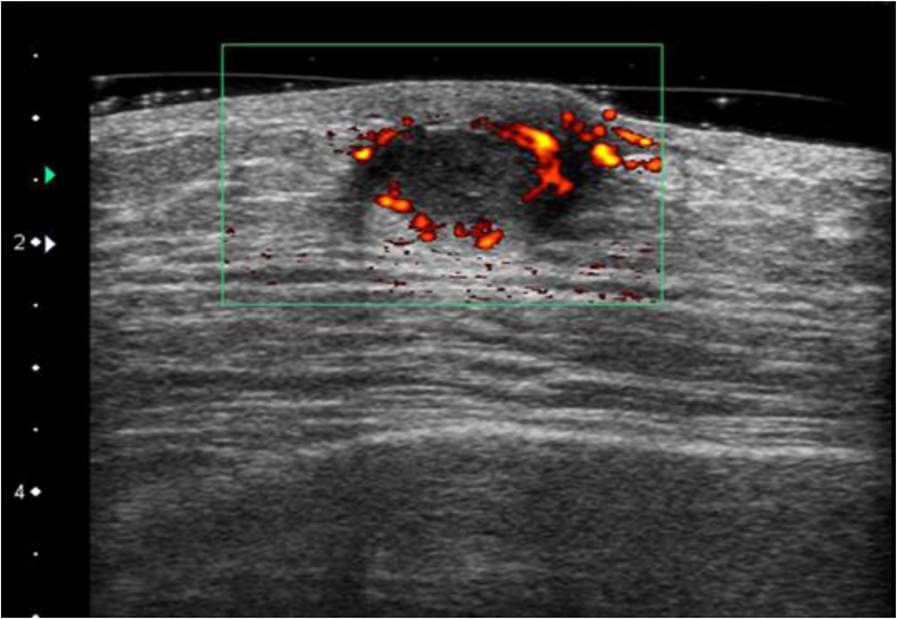
Ultrasonography revealed a hypoechoic lesion 16 mm in size, with accompanying posterior echo attenuation and blood flow. These findings were highly suggestive of a malignant tumor

**Fig. 2:**
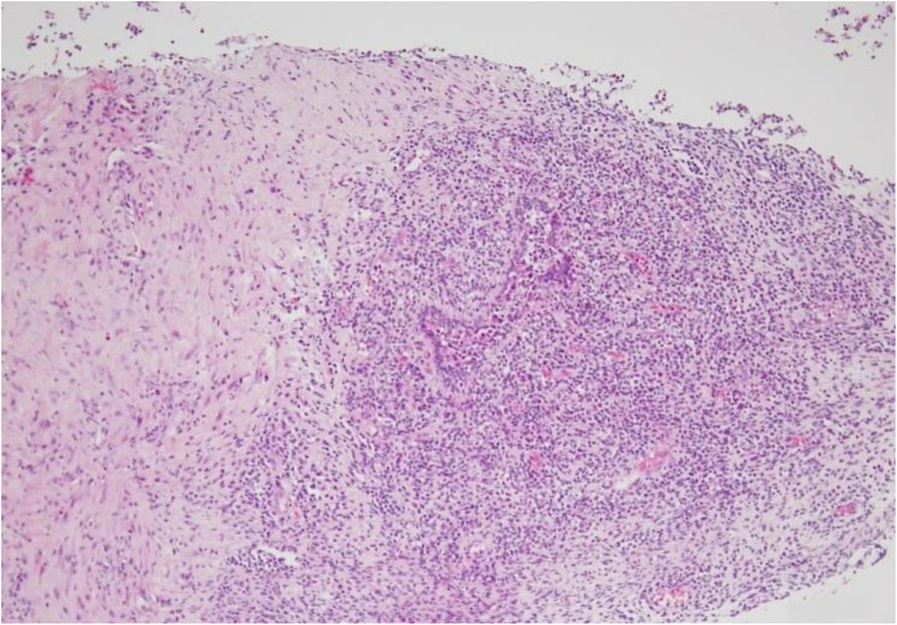
CNB showed lymphocytes and infiltration of primarily inflammatory cells. There was no evidence of malignancy

A 38-year-old man was referred to our institute with a mass in his right breast with nipple discharge. He had no significant medical history. He had a smoking history of 15 cigarettes per day for 15 years. A physical examination revealed an elastic, soft, and mobile mass in the right subareolar region. Ultrasonography revealed a hypoechoic lesion, measuring 16 mm, with accompanying posterior echo attenuation, irregular borders, and blood flow (Fig. 1). Moreover, the internal echo pattern of the mass was heterogeneous. As these findings were highly suggestive of a malignant tumor, an ultrasonography-guided core needle biopsy (CNB) was performed. The CNB findings showed no evidence of malignancy, but they revealed inflammatory cells including lymphocytes (Fig. 2). Based on the above findings, the patient was diagnosed with SBA, and tumorectomy was performed under local anesthesia. Histological examination of the tumor revealed granulation of the tissue with inflammatory changes and a lack of neoplastic changes and evidence of malignancy.

As the abscess recurred 8 months after the operation, the patient underwent an additional complete resection under local anesthesia. The patient has had no recurrence for 1 year and 10 months after the second operation.

### Patient 2

**Fig. 3:**
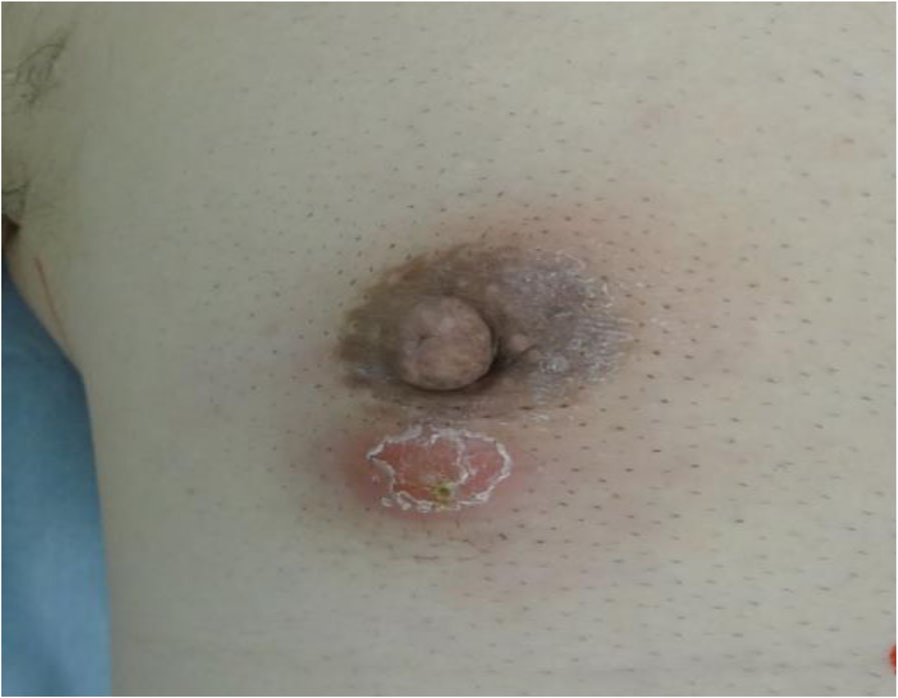
The skin around the nipple was red and inflamed

**Fig. 4:**
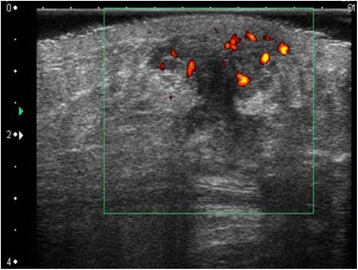
Ultrasonography revealed a hypoechoic lesion 20 mm in size, with border irregularity and blood flow, initially diagnosed malignancy suspected

**Fig. 5:**
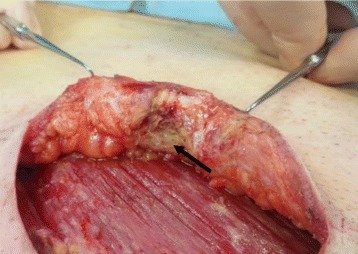
TDR was performed to completely remove the effected ducts and the areolar lesion. The arrow shows the margin of the subareolar mammary duct

**Fig. 6:**
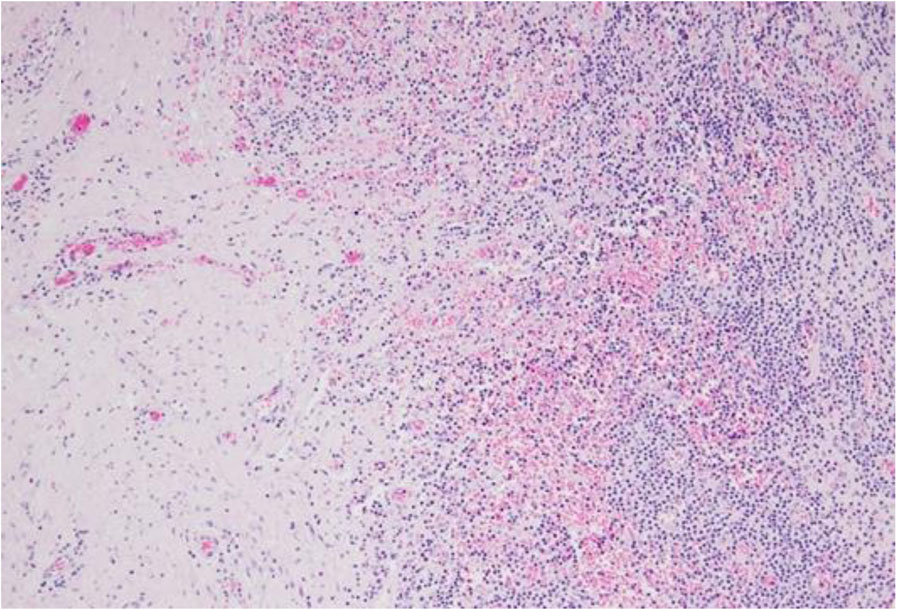
Histopathological diagnosis of the surgical specimen. Neutrophil-based inflammatory cell infiltration and inflammatory granulation of the tissue

A 65-year-old man consulted a local doctor upon noticing a hard mass with skin inflammation in his right breast. At that time, his diagnosis was mastitis and he received an oral antibacterial agent (ampicillin 1500 mg/day) for 14 days. Although the abscess temporarily had shrunk, it grew again 3 months later. He was then referred to our institution for further treatment. His medical history consisted of diabetes, surgery for colon carcinoma, and chronic hepatitis C. He also had a smoking history of 25 cigarettes per day for 40 years. On admission, his physical examination revealed a 20-mm hard mass in the subareolar region. An inflammatory change to the skin on the right lower breast portion under the nipple was observed (Fig. 3). Ultrasonography demonstrated a hypoechoic lesion measuring 20 mm, with an irregular border, blood flow, and a heterogeneous internal echo pattern (Fig. 4). Fine needle aspiration (FNA) showed evidence of an abscess, and the diagnosis was SBA. To obtain curative therapy, tumor and duct resection (TDR) was performed under general anesthesia to remove the effected ducts completely (Fig. 5). The duct tracked from the abscess was identified as the effected duct and was excised along to a nipple as possible. A histological examination revealed neutrophil-based inflammatory cell infiltration and inflammatory granulation of the tissue, which was lacking neoplastic changes (Fig. 6). The diagnosis was consistent with SBA. At present, he has no evidence of recurrence for 2 years and 3 months since the surgery.

### Discussion

SBA was first established as an infection that was distinct from mastitis in 1951 by Zuska et al. [1] regardless of their gender. The pathogenesis of SBA was thought to include the formation of a keratin embolus from the squamous metaplasia of the ductal epithelium; the keratin embolus expands the mammary duct, and an abscess and a fistula were formed by bacterial invasion [2]. On the other hand, follicular obstruction of the pilosebaceous unit had also been pointed out as one of causes; furthermore, there were no unified views on the pathogenesis of a SBA [3–6]. In both patients that we experienced, squamous metaplasia was not observed pathologically. Smoking, diabetes mellitus, obesity, nipple piercing, or nipple inversion has been suggested as a risk factor for SBA [7, 8]. The relationship between smoking and SBA seems particularly strong; Bundred et al. reported that breast abscesses containing anaerobic bacteria were significantly more likely to occur in current smokers than in non-smokers. Both patients were heavy smokers; the abovementioned is likely to apply. Upon diagnosis of this disease, in patient 1, ultrasonography presented findings of malignancy, and CNB was performed. Generally, SBA shows no specific findings [9]. Peifen et al. described the utility of high-resolution magnetic resonance imaging (MRI), which was useful not only for diagnosis but also for identifying landmarks in surgery [10]. Silverman et al. reported that FNA was also useful in the diagnosis of SBA [11]. As for patient 2, the suction of abscess itself leads to the diagnosis of SBA. For the treatment of SBA, total resection of the abscess, including the effected ducts and the formation of an inverted nipple, are the recommended surgical procedures [12–14]. We frequently encounter female cases of SBA, and a large number of cases have been reported in the literature [14]. Breast abscesses are roughly divided into puerperal and nonpuerperal. Most of the nonpuerperal breast abscesses are reported as SBA (86.7% calculated from reference [14]). If the tumor is present under the areola of the nipple, SBA should be taken into consideration. In male patients, diagnoses by image findings are difficult to perform. Therefore, it is necessary to firmly diagnose SBA from malignant tumors with pathological methods such as FNA and CNB. To our knowledge, there have been only nine reports of male patients with SBA. A summary of the characteristics of the previous nine cases and our two patients are shown in Table 1 [11, 15–19]. Most patients were generally under 45 years of age; patient 2 presented herein is an exception to this trend. The disease duration was over 100 days in most cases. FNA was performed in eight cases. CNB was only performed in patient 1 presented herein. All were prediagnosed SBA; five patients underwent resection of their tumors along with the effected ducts. In two cases from the literature, where the disease recurred, both did not have curative operation. For this reason, incisional drainage, which is often performed against breast abscesses, cannot be a curative therapy for SBA. As mentioned above, there is a lack of specific image findings in SBA. In addition, it is difficult to give a diagnosis of SBA only based on the findings of physical examination and imaging test, especially with the two patients we experienced. And we were able to diagnose both cases as SBA before surgery by adding a pathological finding of CNB and FNA. Regarding patient 1, diagnosed as SBA before surgery, TDR was considered. However, due to the patient’s request, he did not prefer TDR. We then decided to carry out a tumor removal procedure under local anesthesia without resection of the effected duct. It has been pointed out that the recurrence rate is high without resection of tumors including effected ducts [14], and this insufficient initial treatment was thus considered to be the cause of recurrence. The two patients we experienced resulted in the contrasting outcomes due to a difference in the surgical procedure, one case recurred and the other did not, which was highly suggestive for future management of SBA.

**Table 1:** Summary of reported cases

Case	Age	DM	SM	DD (day)	DIA (mm)	FNA	CNB	MC	MO	Preoperative diagnosis	Treatment		SQ	Rec
											AB	Surgical method		
1	38			7	40	+	−	+	ND	Breast abscess	+	Incision and drainage		
2	45			365		+	−			Subareolar abscess	+	Resection of tumor including ducts	+	−
3	37			240		+	−			Subareolar abscess	−	NG		
4	42	−		730	10	+	−			Subareolar abscess	−	NG		
5	17			2	10	+	−			Subareolar abscess		Resection of tumor including ducts		
6	41		+	180	10	−	−			Subareolar abscess		Resection of tumor including ducts	+	−
7	39			180	8	−	−			Subareolar abscess	+	Resection of tumor including ducts	−	−
8	45	−		150	15	+	−	+	ND	Subareolar abscess	+	Aspiration drainage		+
9	27			7	100	+	−	+	SA	Subareolar abscess		Incision and drainage		
Patient 1	38	−	+	30	16	−	+	+	ND	Subareolar abscess	−	Simple tumorectomy	−	+
Patient 2	65	+	+	90	20	+	−	−	ND	Subareolar abscess	+	Resection of tumor including ducts	−	−

*DM* diabetes mellitus, *SM* smoking history, *DD* disease duration, *DIA* diameter, *SQ* squamous metaplasia of the surgical specimen, *MC* microbiological culture, *MO* microorganism, *AB* antibiotics, *Rec* recurrence, *ND* not detected, *SA Staphylococcus aureus*, *NG* not given

## Conclusions

Although SBA in male is very rare, it is important to consider it in the differential diagnosis of breast tumors in male patients. Importantly, cytological and pathological analyses are useful for preoperative diagnosis. And to prevent recurrence of the disease, complete resection of the effected duct along with the tumor was recommended essential as derived from previous reports.

## Abbreviations

CNB: Core needle biopsy
FNA: Fine needle aspiration
MRI: Magnetic resonance imaging
SBA: Subareolar breast abscess
TDR: Tumor and duct resection
